# Autologous transplantation of bone marrow mononuclear stem cells by mini-thoracotomy in dilated cardiomyopathy: technique and early results

**DOI:** 10.1590/S1516-31802008000200003

**Published:** 2008-03-06

**Authors:** Renato Abdala Karam Kalil, Daniele Ott, Roberto Sant'Anna, Eduardo Dias, João Pedro Marques-Pereira, Andrés Delgado-Cañedo, Nance Beyer Nardi, João Ricardo Michelin Sant'Anna, Paulo Roberto Prates, Ivo Nesralla

**Keywords:** Dilated cardiomyopathy, Stem cells, Heart surgery, Heart failure, congestive, Cell transplantation, Cardiomiopatia dilatada, Células-tronco, Cirurgia torácica, Insuficiência cardíaca congestiva, Transplante celular

## Abstract

**CONTEXT AND OBJECTIVES:**

There are few studies concerning bone marrow mononuclear cell (BMMC) transplantation in cases of nonischemic dilated cardiomyopathy. This study describes a novel technique of BMMC transplantation and the results up to one year after the procedure.

**DESIGN AND SETTING:**

This was a case series to evaluate the safety and viability of the procedure, at Instituto de Cardiologia do Rio Grande do Sul.

**METHODS:**

Nine patients with symptomatic dilated cardiomyopathy, functional class III/IV and left ventricular ejection fraction (LVEF) < 35% received BMMC (9.6 ± 2.6 x 107 cells) at 20 sites in the ventricular wall, by means of thoracotomy of length 5 cm in the fifth left intercostal space. Echocardiograms and nuclear magnetic resonance (NMR) were performed.

**RESULTS:**

There were no major complications. The functional class results for the first six patients (preoperatively and at two, four, eight and twelve-month follow-ups, respectively) were: [IV-2, III-4] to [I-5, II-1] to [I-3, II-3] to [I-2, II-3] and [I-2, II-3]. Echocardiograms showed LVEF: 25.9 ± 8.2; 32.9 ± 10.4; 29.4 ± 7.2; 25.1 ± 7.9; 25.4 ± 6.8% (p = 0.023); and % left ventricular (LV) fiber shortening: 12.6 ± 4.4; 16.4 ± 5.4; 14.3 ± 3.7; 12.1 ± 4.0; 12.2 ± 3.4% (p = 0.021). LV performance variation seen on NMR was non-significant.

**CONCLUSION:**

Intramyocardial transplantation of BMMC in dilated cardiomyopathy cases is feasible and safe. There were early improvements in symptoms and LV performance. Medium-term evaluation revealed regression of LV function, although maintaining improved functional class.

**CLINICAL TRIAL REGISTRATION NUMBER:**

NCT00615394.

## RESUMO

**CONTEXTO E OBJETIVO:**

Há pouco estudos avaliando o transplante de células mononucleares da medula óssea (CMMO) na miocardiopatia dilatada não-isquêmica. O presente estudo descreve uma técnica de implante intramiocárdico de CMMO por mini-toracomia e resultados com até um ano de acompanhamento.

**TIPO DE ESTUDO E LOCAL:**

Série casos para avaliar segurança e viabilidade do procedimento, no Instituto de Cardiologia do Rio Grande do Sul.

**MÉTODOS:**

Nove pacientes com miocardiopatia dilatada, em classe funcional III/IV e fração de ejeção do ventrículo esquerdo (FEVE) < 35% receberam CMMO (média 9,6 ± 2,6 x 107 células) em 20 pontos da parede livre do ventrículo esquerdo, através de toracotomia de 5 cm no quinto espaço intercostal esquerdo. Foram realizados ecocardiograma e ressonância nuclear magnética (RNM).

**RESULTADOS:**

Não ocorreram complicações maiores. Os resultados pré-operatórios aos 2, 4, 8 e 12 meses de acompanhamento dos seis primeiros pacientes são: classe funcional: IV-2, III-4 para I-5, II-1 para I-3, II-3 para I-2, II-3 e I-2, II-3. Ecocardiograma: FEVE = 25.9 ± 8.2, 32.9 ± 10.4, 29.4 ± 7.2, 25.1 ± 7.9, 25.4 ± 6.8% (p = 0.023); Fração de encurtamento do ventrículo esquerdo (VE) = 12.6 ± 4.4, 16.4 ± 5.4, 14.3 ± 3.7, 12.1 ± 4.0, 12.2 ± 3.4% (p = 0.021). RNM demonstrou diferenças discretas, não significativas.

**CONCLUSÕES:**

O implante intramiocárdico de células-tronco na miocardiopatia dilatada é viável e seguro. Houve melhora precoce nos sintomas e na performance de VE. Avaliação a médio prazo demonstrou regressão da função VE, mantendo, contudo, a melhora na qualidade de vida e na classe funcional.

## INTRODUCTION

Cell transplantation is a promising alternative for promoting cardiac regeneration and improved function.^1^ In experimental models, several types of cells have been used, such as: skeletal myoblasts,^2^ bone marrow mononuclear cells (BMMCs),^3^ mesenchymal stem cells^4^ and cardiomyocytes.^5^ So far, there is no consensus about which type is most suitable. Because BMMCs are easy to obtain and manipulate, they are among the cell types most studied.

As these therapies begin to be transposed to clinical experiments, new inquiries are arising, such as the quantity of cells required, the ambient medium needed and the best administration route. With regard to the latter, several pathways have already been tested in clinical studies: intramyocardial transendocardial, intramyocardial transepicardial, intracoronary and peripheral mobilization through the use of G-CSF (granulocyte colony-stimulating factor).^1^

An ideal approach would involve a minimally invasive method that would be capable of safely injecting the cells intramyocardially, in order to achieve greater local concentrations and fewer systemic effects.

## OBJECTIVE

In this article, our aim is to describe a technique for autologous transplantation of mononuclear bone marrow stem cells, managed by mini-thoracotomy.

## METHODS

Nine patients (six males) of mean age 57.5 ± 9.2 years with a previous diagnosis of nonischemic dilated cardiomyopathy were included. They fulfilled the following selection criteria: (1) diagnosis made more than two years earlier and symptomatic in functional class III-IV according to the New York Heart Association classification (NYHA), despite optimal pharmacological therapy for at least six months prior to inclusion; (2) left ventricular ejection fraction (LVEF) less than 35%, from echocardiogram; (3) age less than 60 years; (4) absence of neoplasm; (5) absence of hematological disease or systemic disease; and (6) no previous cardiac intervention. The exclusion criteria were: (1) episodes of tachycardia or ventricular fibrillation; (2) severe or moderate mitral insufficiency; and (3) any other valve diseases.

The preoperative evaluation included: Doppler echocardiogram, myocardial nuclear magnetic resonance, six-minute walk test, quality-of-life evaluation using the Minnesota Living With Heart Failure questionnaire, electrocardiogram, hemogram, electrolytes, chest x-ray and clinical evaluation. The patients included were selected for two studies: an initial pilot series of six cases to evaluate the safety and viability of the procedure, followed by a randomized clinical trial, which is now in progress, and in which three new cases have so far been included.

The project was approved by the local and the national ethics committees in the year 2004, under number 3549, and has been awarded a grant from FAPERGS (State of Rio Grande do Sul Research Foundation).

### Stem cell preparation

**Figure 1:**
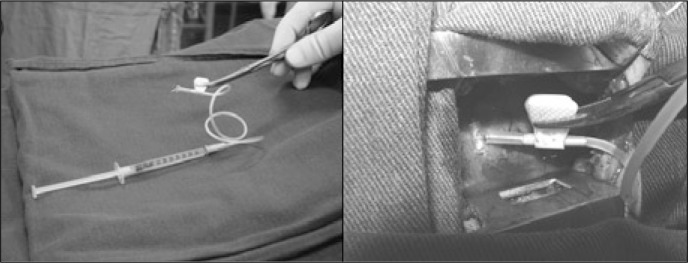
Left: cell suspension ready for use. Right: Intramyocardial injection of mononuclear cells.

Approximately four hours before the operation and with the patient under sedation, bone marrow was collected by aspiration puncture in the anterosuperior iliac crest, obtaining a total aspired volume of 80 ml, in a medium containing anticoagulant preservative (Figure 1). The bone marrow mononuclear stem cells were isolated by centrifugation using Ficoll-Hypaque density gradient medium (1.077 g/ml). The mononuclear fraction was then collected, washed in a heparinized saline solution containing 5% autologous serum and filtered in order to remove the cellular adjunctive. The cells were again suspended in saline solution with autologous serum at 5% concentration, to make up an intramyocardial injection volume of 5 ml. A small fraction was utilized for sterility tests, cell counting and viability tests. Viability greater than 90% was considered acceptable. Another fraction of the cells was used for immunophenotyping using flow cytometry, in order to determine the subpopulations (CD 34^+^, CD 38^+^ and subpopulations of lymphocytes and monocytes) and to carry out functional analysis.

### Surgical technique

The surgical approach was through left mini-thoracotomy, consisting of an incision of approximately 5 cm in length, anterolaterally in the fifth left intercostal space (Figure 1), in order to expose the pericardium. A T-shaped pericardial incision was made and 2-0 polyester traction sutures were placed. Pericardial fluid was suctioned and the free wall of the left ventricle was exposed. Videothoracoscopy equipment for viewing was used only in the first patient. For subsequent patients, it was left available in the operating room but not used, because it was considered unnecessary due to the proximity of the heart to the thoracic wall in these patients. Once the position of the coronary arteries had been defined, the injections of the cell suspension were made directly, through a 21F butterfly needle that was positioned in the myocardium and connected to an extension managed by the surgical assistant.

**Figure 2:**
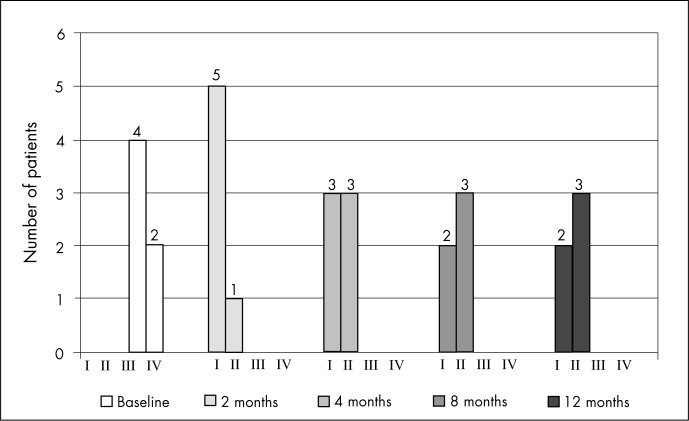
Patients’ functional class during the twelve months of follow-up.

Twenty small injections in the myocardium were made, with a total volume of 5 ml or about 0.25 ml per injection site, in the anterior, lateral, posterior and apical faces of the left ventricle, containing a mean total of 9.6 ± 2.6 x 10^7^ cells (Figure 2). After reviewing the hemostasis, the pericardium was closed with a single suture of 2-0 polyester, the thoracic cavity was drained and the chest wall was closed. After the procedure, the patients were kept in the postoperative intensive care unit for a minimum period of 24 hours. They were released from the hospital after a time ranging from five to seven days.

## RESULTS

### Surgical procedure

No major complications were observed. During the procedure, one patient developed ventricular arrhythmia because the heart was touched, which impeded adequate expose for injections. The arrhythmia ceased completely after endovenous lidocaine administration and the procedure was then completed. In another case, it was necessary to place hemostatic sutures at two sites in the ventricular wall where injections of the cell suspension were applied, due to slight but persistent bleeding that did not cease with prolonged compression.

The duration of the bone marrow collection procedure was 69 ± 31 minutes. The mean duration of the thoracotomy procedure was 105 ± 23 minutes. After the procedures, the patients remained under observation in the intensive care unit. There were no significant postoperative complications. There were no readmissions during the follow-up period due to heart failure.

### Clinical results

#### Safety and hospital admission

One patient returned to the emergency room during the first postoperative month, vaguely complaining of malaise and anxiety. He was examined and did not present signs of cardiac failure, infection or postoperative complications. Seven months later, this patient presented a lower limb abscess and bacteremia that led to septic shock and death in an emergency room at another hospital. No postmortem examination was carried out.

#### Cell analysis

Cell analyses were performed on the first five patients. The cell viability was greater than 99%, thus vouching for the cell suspension quality. Fungal and bacterial cultures were negative. A mean total of 9.6 ± 2.6 x 107 cells was injected into each patient. The CD 34+ fraction represented 1.5 ± 0.7%. Other cell fractions identified were: CD 45+ (74.6 ± 8.5%), CD 14+ (8.4 ± 4.7%), CD 34+ CD 38- (0.7 ± 0.5%) (Table 1).

**Table 1.** Immunophenotyping of bone marrow mononuclear cells administered to patients (frequency of cells for each marker)

Patient	CD45+*	CD14+†	CD3+ CD4+*	CD3+ CD8+*	CD34+†	CD34+ CD38†	Number of cells injected
1	82.0	13.0	27.0	17.0	0.6	0.2	7.0 × 10^7^
2	81.0	14.0	27.0	10.0	0.9	0.3	1.3 × 10^8^
3	61.0	5.0	17.0	2.0	2.0	1.2	7.0 × 10^7^
4	77.0	6.0	19.0	3.0	1.6	ND	1.0 × 10^8^
5	72.0	4.0	24.0	9.0	2.6	1.1	1.1 × 10^8^
Mean	74.6	8.4	22.8	8.2	1.5	0.7	9.6 × 10^7^
SD	8.6	4.7	4.6	6.1	0.8	0.52	2.6 × 10^7^

* Percentage of cells in relation to total number of viable cells;

† Percentage of cells in relation to CD45+ cells.

SD = standard deviation.

#### Clinical results

**Figure 3:**
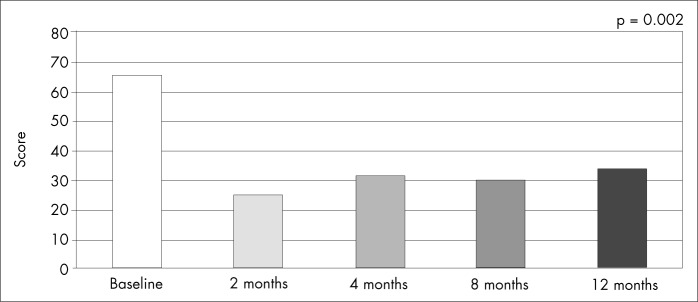
Quality of life of the patients during the 12 months of follow-up, from Minnesota Living With Heart Failure questionnaire.

All the patients presented clinical improvement, as evaluated by the NYHA functional class (FC). At the baseline evaluation, four patients were in FC III and two were in FC IV. At the two-month follow-up, five patients were asymptomatic and one was in FC II. After one year of follow-up, two of the patients remained asymptomatic and three were in FC II (p = 0.001) (Figure 2). This clinical improvement was corroborated by the Minnesota Living with Heart Failure questionnaire, which showed significant improvements in quality of life, expressed as reductions in overall scores over the course of the twelve months of follow-up (p = 0.002) (Figure 3).

**Figure 4:**
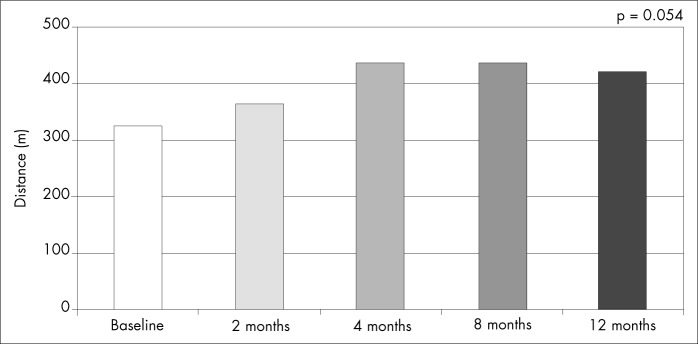
Six-minute walk test on the patients during the twelve months of follow-up.

Functional capacity also showed a tendency towards improvement after four months of follow-up, and for the remaining of the follow-up period, as evaluated by the six-minute walk test, although not reaching statistical significance (Figure 4).

### Left ventricular (LV) performance

**Figure 5:**
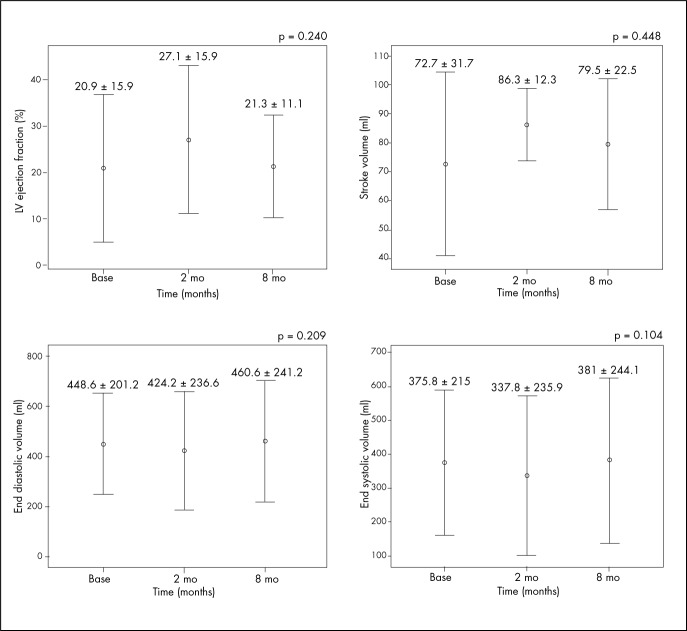
Nuclear magnetic resonance results for left ventricular functional parameters.

Nuclear magnetic resonance evaluation at the two-month follow-up showed reductions in end systolic volume (ESV) and end diastolic volume (EDV), along with increased stroke volume (Table 2). As a result, the LVEF showed an early absolute increase of 6.2% and relative increase of 45.5% (20.9 *±* 15.9% to 27.1 *±* 15.9%; p = 0.249). At the eight-month follow-up, these parameters showed decreases towards baseline values, but a relative increase of 24.4% was still observed for LVEF. The ventricular mass was not modified (Table 2) (Figure 5).

**Table 2.** Absolute values for evaluation of left ventricular performance by means of nuclear magnetic resonance

	Baseline Mean ± SD	Two months Mean ± SD	Eight months Mean ± SD	p*
Left ventricular ejection fraction (%)	20.9 ± 15.9	27.1 ± 15.9	21.3 ±11.1	0.249
Stroke volume (ml)	72.7 ± 31.7	86.3 ± 12.3	79.5 ± 22.5	0.448
Left ventricular end diastolic volume (ml)	448.6 ± 201.2	424.2 ± 236.6	460.6 ± 241.2	0.209
Left ventricular end systolic volume (ml)	375.8 ± 215	337.8 ± 325.9	381 ± 244.1	0.104

* p = for repeated-measurement analysis of variance (ANOVA) adjusted according to Bonferroni; SD = standard deviation.

**Figure 6:**
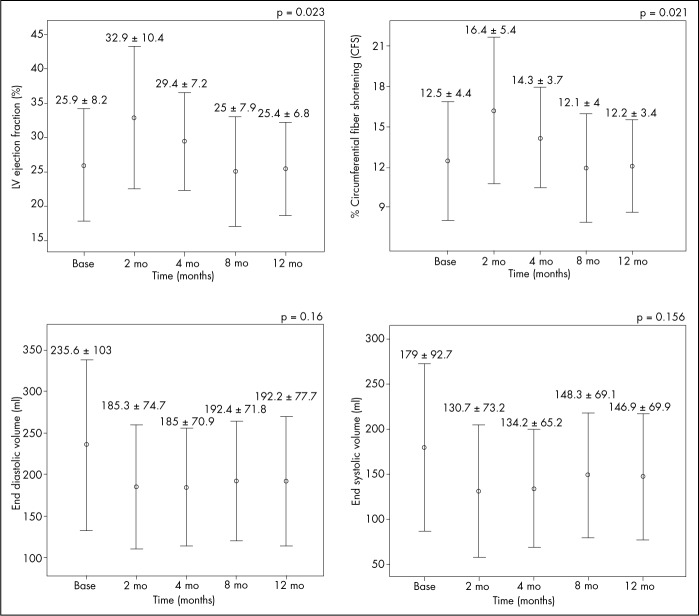
Echocardiogram results for left ventricular evaluation.

Improved ventricular function was shown by Doppler echocardiography (Table 3 and Figure 6). LVEF showed statistically significant differences, with an absolute increase of 6.93% and relative increase of 29.6% (25.9 *±* 8.2% x 32.9 *±* 10.4%; p = 0.023 and p = 0.012, respectively) at the two-month follow-up. A slight decrease in LVEF was observed at the four-month follow-up (29.4 *±* 7.2%), and the results at the eight and twelve-month follow-ups (25.4 *±* 6.8%) were similar to baseline values. Increased circumferential fiber shortening and reduced ESV and EDV were seen at the two-month follow-up; these values decreased over the next six months and showed a tendency towards stabilization at the eight and twelve-month postoperative assessments. The echo imaging also showed a reduction in ventricular mass (226.2 ± 41.0 to 214.05 ± 54.9 g after twelve months), which did not reach significance level (p = 0.44).

**Table 3.** Absolute values for left ventricular performance, by means of echocardiogram

	Baseline Mean ± SD	Two months Mean ± SD	Four months Mean ± SD	Eight months Mean ± SD	One year Mean ± SD	p*
Left ventricular ejection fraction (%)	25.9 ± 8.2	32.9 ± 10.4	29.4 ± 7.2	25.6 ± 7.9	25.4 ± 6.8	0.023
Left ventricular circumferential fiber shortening (%)	12.6 ± 4.4	16.4 ± 5.4	14.3 ± 3.7	12.1 ± 4.0	12.2 ± 3.4	0.021
End diastolic volume (ml)	235.6 ± 103	185.3 ± 74.7	185 ± 70.9	192.4 ± 71.8	192.2 ± 77.7	0.163
End systolic volume (ml)	179 ± 92.7	130.7 ± 73.2	134.2 ± 65.2	148.3 ± 69.1	146.9 ± 69.9	0.156

* p = for repeated-measurement analysis of variance (ANOVA) adjusted according to Bonferroni; SD = standard deviation.

**Figure 7:**
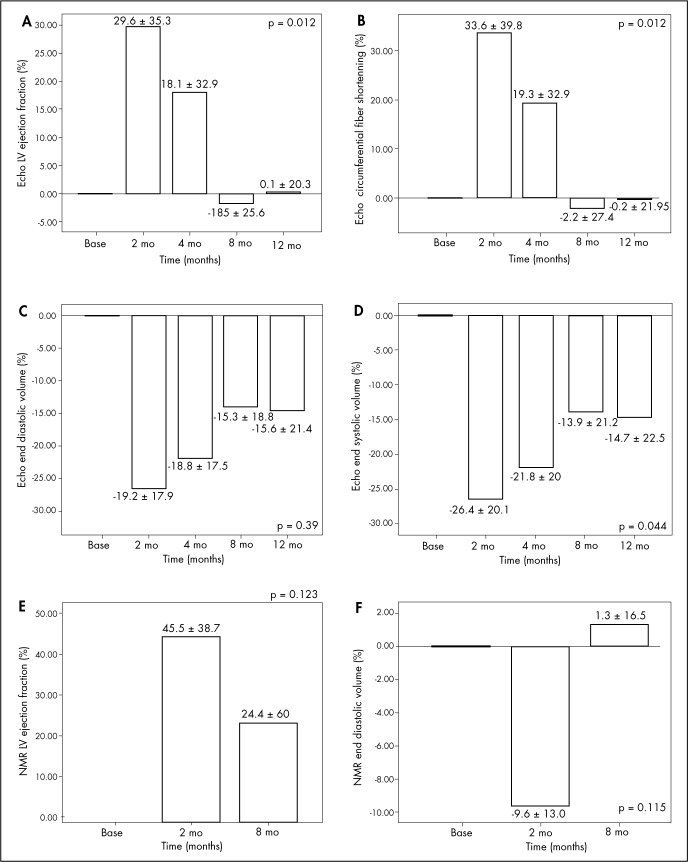
Percentage variation relative to baseline (preoperative measurements) for left ventricular parameters, estimated by echocardiogram (echo) and nuclear magnetic resonance (NMR). A = left ventricular ejection fraction (echo). B = left ventricular circumferential fiber shortening (echo). C = end diastolic volume (echo). D = end systolic volume (echo). E = left ventricular ejection fraction (NMR). F = left ventricular end diastolic volume (NMR).

Figure 7 presents the variation (increases or decreases) in LV performance assessment values from echocardiograms and nuclear magnetic resonance, expressed as percentages of the baseline (preoperative) levels. The most significant findings were a relative and temporary improvement of up to 29.6% in LVEF on echocardiogram at the two-month follow-up (A); a 33.6% improvement in circumferential fiber shortening at the same time (B) and a similar relative increase in LVEF from NMR evaluation (45.5%) (E). Table 4 shows the percentage increases or decreases in left ventricular echocardiogram and NMR values, demonstrating the variations relative to preoperative baseline measurements (Table 4).

**Table 4.** Percentage increase or decrease in left ventricular echocardiogram and NMR values, demonstrating the variations relative to preoperative baseline measurements

	Two months	Four months	Eight months	One year	p*
	Mean ± SD	Mean ± SD	Mean ± SD	Mean ± SD	
	%	%	%	%	
Echocardiogram					
Ejection fraction	29.6 ± 35.3	18.1 ± 32.9	-1.85 ± 25.6	0.1 ± 20.3	**0.012**
Circumferential fiber shortening	33.6 ± 39.8	19.3 ± 32.9	-2.2 ± 27.4	-0.2 ± 21.9	**0.01**
End diastolic volume	-19.2 ± 17.9	-18.8 ± 17.5	-15.3 ± 18.8	-15.6 ± 21.4	0.39
End systolic volume	-26.4 ± 20.1	-21.8 ± 20.0	-13.9 ± 21.2	-14.7 ± 22.5	**0.044**
NMR					
Ejection fraction	45.5 ± 38.7	-	24.4 ± 60.0	-	0.123
End diastolic volume	-9.6 ± 13.0	-	1.3 ± 16.5	-	0.115

* p = for repeated-measurement analysis of variance (ANOVA) adjusted according to Bonferroni; NMR = nuclear magnetic resonance; SD = standard deviation.

## DISCUSSION

Our experience indicated that intramyocardial stem cell transplantation in patients with nonischemic dilated cardiomyopathy, by means of mini-thoracotomy, was feasible, safe, well-tolerated and associated with initial clinical improvement, as expressed by better functional capacity and ventricular function in the immediate postoperative period. However, this improvement in ventricular function was lost after four months of follow-up, with progressive deterioration of some parameters, which returned to levels similar to the preoperative values. No major procedure-related complications occurred. There was a relatively prolonged length of hospital stay, until the patients achieved thorough recovery after the procedure. This was certainly longer than hospital stays relating to less invasive procedures. Nevertheless, early hospital discharge was not of concern in this initial series.

This method of surgical transplantation of bone marrow mononuclear stem cells has potential advantages: (1) application in the myocardium under direct viewing, which allows precise and safe injection; (2) low complexity and low cost, making it available to almost all cardiac surgical centers; (3) minimal dispersion of cell solution, thus minimizing systemic effects. The potential drawbacks would be: (1) the need for thoracotomy, with its related morbidity; (2) using small incisions, especially in patients with significant cardiac dilatation, it may be difficult to reach the entire free wall of the LV; (3) the ventricular septal myocardium cannot be directly approached.

Clinically, the types of therapy^1^ that bone marrow stem cells have most been used for are intracoronary infusion^6^, transepicardial intramyocardial infusion during cardiac surgery^7^, transendocardial intramyocardial infusion in patients for whom revascularization using the NOGA System was not possible^8^ and peripheral mobilization by means of G-CSF.^9^ Generally speaking, the intracoronary route would be beneficial, since it is less invasive and less expensive, although it would have the drawback of not applying the cells directly to the lesioned tissue and would therefore depend on a homing process. One other disadvantage of intracoronary injection would be the fact that cells tend to migrate to systemic circulation, which could theoretically accelerate diseases dependant on neovascularization, such as diabetic retinopathy and neoplasms.

In patients with chronic ischemia, the transendocardial intramyocardial approach by catheterization has been preferred, using the NOGA navigation system.^6^ This method not only enables intramyocardial application less invasively, but also allows mapping of myocardial areas such that injection becomes more viable. Its drawbacks are that it presents little system availability and high cost. In specific clinical experiments, its use has been associated with clinical symptomatic and LV function improvements that were attributable to better myocardial perfusion.

Peripheral mobilization of bone marrow cells using G-CSF has not been associated with favorable results in several randomized clinical trials among patients with heart failure, or has only been associated with very small benefits. Different results had been obtained in experimental studies. Thus, this approach seems to be less effective.

There are no published complete original studies using bone marrow cells in patients with nonischemic dilated cardiomyopathy. A successful clinical series of patients with Chagas disease on whom intracoronary infusion was used has recently been published.^10^ Trainini et al.^11^ evaluated the effects in eight cases of nonischemic dilated cardiomyopathy. No procedure-related complications occurred. Over a mean follow-up period of 295 ± 37 days, they found a significant improvement in NYHA functional class (2.5 ± 0.8 to 1.5 ± 0.5) and LVEF (18.3 ± 7% to 26.4 ± 10%).^11^

In our experience, we found it unnecessary to use thoracoscopy equipment, because the proximity between the heart (especially when dilated) and the chest wall made the procedure completely achievable, in safety, through a small incision that was similar to the one utilized for epicardial electrode implants for pacemakers.

The main limitations of this study were: (1) the relatively small sample size: (2) the uncontrolled phase I study design, such that the improvements observed could be at least partly due to better adherence by the patients to the treatment; and (3) although the follow-up period used in the present study was one of the longest among cell therapy clinical trials for nonischemic dilated cardiomyopathy, it did not allow conclusions regarding the long-term effects.

Progressive improvement in heart function was observed for two months, with a slight decline during the following 10 months of follow-up. The clinical situation as a whole showed a slight late worsening (Figure 2), but some degree of recovery was maintained and none of the patients returned to functional class III or IV during the twelve months of follow-up. Major responses to the therapy were seen in ejection fraction and end systolic volume.

It is possible that the temporary nature of the improvement in ventricular function observed in the present study occurs more generally and was not reported by others because most investigations have been restricted to short and medium-term follow-up of a few weeks. In some studies, such as the one reported by Perin et al.^8^, however, sustained positive results from cell therapy for ischemic heart failure were reported after six and twelve-month follow-up periods. In a randomized study with transplantation of BMMCs in cases of acute infarction, Meyer et al.^12^ observed improvements six months after treatment but, at the 18-month follow-up, the conditions of the treated and control patients did not show any significant differences.

All of our patients, even those for whom the objective evaluation did not show improved ventricular function, reported subjective improvement following cell therapy. The placebo effect cannot be ignored in this kind of approach, and it may result in better adherence to treatment. Even without a control group, however, the results in general show significant improvement in heart function, particularly considering that the inclusion criteria involved at least two years since diagnosis and complete treatment for at least six months, conditions under which progressive clinical deterioration would be expected.

## CONCLUSIONS

In conclusion, the described technique of mini-thoracotomy in the fifth left intercostal space, even without the aid of videothoracoscopy, is feasible and safe, with low surgical risks. Therefore, this may be a suitable approach for accessing the free wall of the LV to inject planned quantities of bone marrow stem cells, in cases of nonischemic dilated cardiomyopathy. There were early improvements in symptoms, quality of life and LV performance. Medium-term evaluation revealed regression of LV function nearly to preoperative levels, although improved functional class and quality of life were maintained.
